# Hypoxia preconditioned bone marrow mesenchymal stem cells promote liver regeneration in a rat massive hepatectomy model

**DOI:** 10.1186/scrt234

**Published:** 2013-07-15

**Authors:** Jun Yu, Shengyong Yin, Wu Zhang, Feng Gao, Yuanxing Liu, Zhiyun Chen, Min Zhang, Jiangjuan He, Shusen Zheng

**Affiliations:** 1 Department of Hepatobiliary and Pancreatic Surgery, The First Affiliated Hospital, School of Medicine, Zhejiang University, Key Laboratory of Multi-Organ Transplantation of Ministry of Public Health, Hangzhou 310003, China

**Keywords:** Bone marrow mesenchymal stem cell, Liver regeneration, Hypoxia preconditioning

## Abstract

**Introduction:**

Bone marrow mesenchymal stem cells (BMMSCs) have been reported to facilitate liver regeneration after toxic injuries. However, the effect of BMMSCs on liver regeneration after massive hepatectomy is barely studied. Here we explored whether infusion of BMMSCs promotes liver regeneration in a rat massive hepatectomy model.

**Methods:**

Hypoxia preconditioning was achieved by culturing BMMSCs under a hypoxia environment. Then 85% hepatectomy was performed and hypoxia or normoxia preconditioned BMMSCs were infused into the portal vein. A group of rats received vascular endothelial growth factor (VEGF) neutralizing antibody perioperatively, and underwent 85% hepatectomy and a subsequent infusion of hypoxia preconditioned BMMSCs to verify the role of VEGF in the effects of BMMSCs on liver regeneration. Liver samples were collected and liver regeneration was evaluated postoperatively.

**Results:**

Hypoxia preconditioning enhanced the expression of VEGF in BMMSCs *in vitro*. Infusion of BMMSCs promoted proliferation of hepatocytes, reflected by elevated cyclin D_1_ expression and proliferating cell nuclear antigen-positive hepatocytes. However, BMMSC infusion did not improve the serum albumin level, liver weight/body weight ratio, and survival after operation. Infusion of hypoxia preconditioned BMMSCs significantly elevated cyclin D_1_, proliferating cell nuclear antigen-positive hepatocytes, liver weight/body weight ratio, and survival compared with normoxia preconditioned BMMSCs, accompanied by an increased serum albumin level. The level of VEGF in liver homogenate was much higher in hypoxia preconditioned BMMSC-treated animals than in other groups. In addition, the perioperative injection of VEGF neutralizing antibody significantly blocked the therapeutic effects of hypoxia preconditioned BMMSCs on liver injury and regeneration in this model.

**Conclusion:**

Hypoxia preconditioned BMMSCs enhanced liver regeneration after massive hepatectomy in rats, possibly by upregulating the level of VEGF.

## Introduction

The liver is the only human organ that is capable of natural restoration of lost tissue, mainly by proliferation of previously quiescent hepatocytes. As little as 25% of a liver is reported to be able to regenerate into a whole liver in a short period. However, in some severe conditions, such as acute liver failure or end-stage liver diseases, the proliferation of hepatocytes is insufficient or seriously impaired, leading to failure of liver function. At present, the only therapeutic choice for acute liver failure and end-stage liver diseases is liver transplantation. However, as long as the donor shortage continues to be the rate-limiting step in the expansion of liver transplantation, work on exploring alternative forms of therapies is still needed.

Stem cell-based therapy is emerging to be an effective treatment in many diseases. Adult mesenchymal stem cells (MSCs) have recently received attention for their availability and ability to differentiate down multiple lineages [1,2]. Bone marrow mesenchymal stem cells (BMMSCs) are one type of MSCs derived from bone marrow and are considered a promising cell source in treating various liver disorders [3-5]. Systemic infusion of BMMSCs into rodents subjected to toxic liver injuries is confirmed to restore liver function and promote liver regeneration [5]. Some clinical trials also indicate that infusion of BMMSCs benefits patients with end-stage liver diseases [6-8].

Although BMMSCs have been shown to attenuate liver injuries and promote liver regeneration, the underlying mechanisms are still not clear. Transdifferentiation into the hepatic lineage is not considered a convincing explanation because there is no solid evidence. Cell fusion may better explain the coexpression of hepatic and MSC markers [9,10]. On the other hand, the paracrine effect of BMMSCs is drawing increasing attention and is thought to be the main mechanism of its therapeutic effects. MSC-conditioned medium therapy has been reported to provide trophic support to the injured liver by inhibiting hepatocellular death and stimulating regeneration [11], highlighting the role of MSC-derived soluble factors in attenuating liver injury and promoting liver regeneration. BMMSCs secrete many growth factors, cytokines and chemokines, including vascular endothelial growth factor (VEGF), hepatocyte growth factor, IL-6, and stromal cell-derived factor-1 [12]. Among these factors, VEGF is an important growth and proangiogenic factor, and is regarded as the key factor secreted by BMMSCs in treating many diseases [13-16]. VEGF is also considered an important factor during liver regeneration [17,18]. BMMSCs might therefore promote liver regeneration by secreting VEGF and other trophic factors. BMMSCs are reported to produce more growth factors under a hypoxic environment [19], and hypoxia preconditioning is confirmed to be an effective way to enhance their therapeutic potential [20,21]. Hypoxia preconditioning of BMMSCs might therefore enhance their effect on liver regeneration by upregulating the secretion of VEGF.

Here we explored whether infusion of normoxia or hypoxia preconditioned BMMSCs promotes liver regeneration in a rat 85% hepatectomy model. The role of BMMSC-derived VEGF was also evaluated by measuring the cells’ expression *in vitro* and *in vivo.*

## Materials and methods

### Isolation and culture of BMMSCs

BMMSCs were isolated from rat femoral bone marrow. In brief, the marrow pellet was washed in Hanks’ balanced salt solution, centrifuged at 1,000 rpm for 10 minutes and then resuspended in DMEM (Gibco, Grand Island, NY, USA) containing 10% fetal bovine serum (Gibco), penicillin (100 U/ml), and streptomycin (100 mg/ml). The nucleated cells were cultured at 37°C in a humidified 5% CO_2_ atmosphere. The medium was replenished 24 hours later to remove the nonadherent cells and every 3 days thereafter. For subculture, cells were resuspended with 0.25% trypsin and passaged at a ratio of one to three plates.

**Figure 1:**
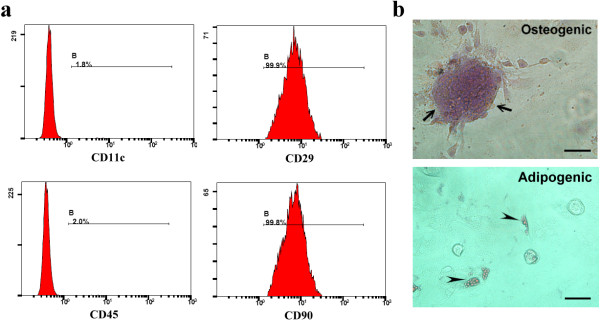
**Expression of several surface markers and multi-potential of bone marrow mesenchymal stem cells. (a)** Most of the isolated bone marrow mesenchymal stem cells (BMMSCs) expressed CD29 and CD90, few expressed CD11c and CD45. **(b)** Matrix mineralization (arrows) or lipid droplets (arrow heads) were seen in cells cultured in osteogenic or adipogenic medium for 4 weeks. *n* = 6, scale bar represents 10 μm.

For analysis of cell surface markers, passage 3 cells were incubated with fluorescein isothiocyanate-conjugated mouse antibodies against rat CD11c, CD29, CD45, and CD90 (Caltag Laboratories, San Francisco, CA, USA) and analyzed by flow cytometry. Most of these cells expressed CD29 and CD90, and few expressed CD11c and CD45 (Figure 1a).

The multipotency of isolated cells was evaluated using adipogenic and osteogenic assays. Briefly, passage 3 cells were cultured in DMEM containing 10% fetal bovine serum, antibiotics, 200 mM indomethacin, 1 mM dexamethasone, 0.5 mM 3-isobutyl-1-methylxanthine and 10 g/l insulin (Sigma-Aldrich, St Louis, MO, USA) for adipogenic induction or in DMEM containing 10% fetal bovine serum, antibiotics, 50 mg/l l-ascorbate-2-phosphate, 0.1 μM dexamethasone, 10 mM β-glycerophosphate(Sigma–Aldrich) for osterogenic induction. Four weeks later, oil Red O and alizarin red stainings were performed to detect the lipid droplets and matrix mineralization in induced cells respectively (Figure 1b).

### Hypoxia-preconditioning of BMMSCs

Passage 3 to 5 BMMSCs were suspended using 0.25% trypsin and plated into new dishes in 5 × 10^5^/ml. The media was refreshed 24 hours later and cells were cultured under 1% oxygen, 5% CO_2_, and 94% nitrogen for another 24 hours. After 24 hours’ hypoxic culture, these cells were immediately harvested and resuspended in saline (5 × 10^6^ cells/ml) prior to infusion into animals. The levels of VEGF in the supernatant were measured using a rat VEGF ELISA kit (R&D systems, Minneapolis, MN, USA), following the manufacturer’s protocol. The experiment was repeated three times. At least six plates per experiment were used in each group.

### Experimental design and rat 85% hepatectomy

Male Sprague–Dawley rats were purchased from Shanghai Laboratory Animal Center, Chinese Academy of Sciences. The animals were housed in a controlled 12-hour light/dark cycle environment with chow and water *ad libitum*. The animal experiments were performed according to guidelines for animal care of Zhejiang University and approved by the Animal Ethics Review Committees of Zhejiang University.

A total of 156 rats were used in this study. They were divided into four groups: control group (*n* = 24), rats underwent 85% hepatectomy and received 1 ml saline; N-BMMSC group (*n* = 24), rats underwent 85% hepatectomy and received 5 × 10^6^ normoxia-conditioned BMMSCs in 1 ml saline; H-BMMSC group (*n* = 24), rats underwent 85% hepatectomy and received 5 × 10^6^ hypoxia-conditioned BMMSCs in 1 ml saline; and H-BMMSC + VEGF mAb group (*n* = 24), rats underwent 85% hepatectomy, received 5 × 10^6^ hypoxia-conditioned BMMSCs in 1 ml saline, and were injected (intraperitoneally) with VEGF mAb (100 μg/mouse) six times perioperatively. Another 15 rats from each group were used for the survival study.

The 85% hepatectomy was performed according to the published method [22]. Briefly, after general anesthesia with sodium pentobarbital (30 to 50 mg/kg, intraperitoneally), rat middle lobes, left lateral lobe, caudate lobes and right inferior lobe were ligated and resected. Subsequently, 1 ml saline with or without 5 × 10^6^ BMMSCs was infused into the portal vein. After operation, rats were allowed to spontaneously recover, and no further treatment was given. The hepatectomy was performed by an experienced technician. All animals survived the surgical procedure in this study. Rats were sacrificed 24, 36, 72, and 168 hours after operation. Liver weight and body weight were measured. Serum and liver samples were collected for analysis.

### Real-time RT-PCR

Total mRNA was extracted from frozen tissue samples by Trizol (Invitrogen, Carlsbad, CA, USA) and reverse transcribed using an RT reagent kit (TaKaRa, Shiga, Japan). Real-time PCR was performed using an ABI 7900 Sequence Detection System (Applied Biosystems, Foster City, USA) with SYBR-green I (TaKaRa). The primer sequences were listed as follows: cyclin D_1_ – sense, 5′-CTTCAAGTGCGTGCAGAGGGAG-3′ and anti-sense, 5′-GTAGTTCATGGCCAGCGGGAAG-3; VEGF – sense, 5′-TGCACTGGACCCTGGCTTTAC-3′ and anti-sense, 5′-CGGCAGTAGCTTCGCTGGTAG-3′; and β-actin – sense, 5′-GAAGAGCTATGAGCTGCCTGAC-3′ and anti-sense, 5′-AGGTCTTTACGGATGTCAACGT-3′. Relative gene expression profiles were determined by normalizing to the housekeeping gene (β-actin) using the 2^−(ΔΔCt)^ method [23].

### Western blot

Total proteins were extracted in cell lysis buffer with protease and phosphatase inhibitors. Proteins were quantified using protein assay (Bio-Rad Laboratories, Hercules, CA, USA), and 50 mg protein was separated by SDS-PAGE and transferred to 0.2 mm polyvinyl difluoride membranes. Membranes were blocked in Tris-buffered saline, 0.1% Tween 20, and 5% BSA for 2 hours before overnight incubation with polyclonal rabbit antibodies against rat cyclin D_1_ and VEGF (diluted 1:2,000 in Tris-buffered saline 0.1% Tween 20 and 5% BSA; Abcam, Cambridge, MA, USA). The membranes were incubated for 1 hour in horseradish-peroxidase-conjugated goat anti-rabbit IgG (eBioscience, San Diego, CA, USA) at a dilution of 1:2,000 in Tris-buffered saline, 0.1% Tween 20 and 5% BSA. Immunoreactive protein was detected using ECL (GE Healthcare, Chalfont St Giles, UK) and BioMaxfilm (Kodak, Rochester, NY, USA).

### Immunohistological assay

Paraffin-embedded liver sections were stained for proliferating cell nuclear antigen (PCNA). Liver slides were stained with rabbit polyclonal antibody to PCNA (1:100; Santa Cruz Technology, Santa Cruz, CA, USA) at 4°C overnight. The sections were incubated with second antibodies and stained with chromogenic substrate solution. The amount of PCNA-positive cells was counted by two independent and blinded reviewers. Briefly, they calculated the PCNA-positive cells (with brown nuclei) of four independent microscopic fields (×40) per section and two sections per animal.

### ELISA of liver homogenate for VEGF

Snap-frozen liver samples were thawed on ice, weighed, and homogenized in solution containing 2 mg protease inhibitor (Sigma-Aldrich) per milliliter of normal saline. The expression of VEGF in liver homogenate was evaluated by ELISA according to the manufacturer’s protocol.

### Statistical analysis

Data were analyzed using SPSS 16.0 software (SPSS, Chicago, IL, USA). One-way analysis of variance was used to compare group variables, followed by Tukey’s *post-hoc* testing when indicated. Games–Howell testing was performed for distributions for which equal variances could not be assumed. All values were expressed as mean ± standard deviation. *P* <0.05 was considered significant. To analyze the 7-day survival rate, the Kaplan–Meier method and log-rank test was applied.

## Results

### Hypoxia preconditioning enhanced the secretion of VEGF by BMMSCs

**Figure 2:**
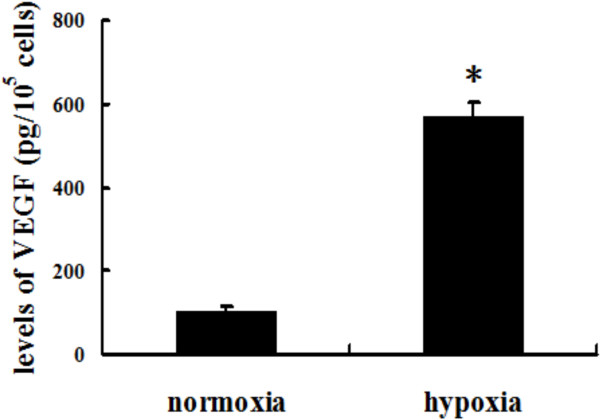
**Vascular endothelial growth factor in bone marrow mesenchymal stem cells incubated under normoxia or hypoxia.** Levels of vascular endothelial growth factor (VEGF) in supernatant of bone marrow mesenchymal stem cells incubated under normoxia or hypoxia conditions for 24 hours. *n* = 6, *P <0.05.

To explore whether the secretion of VEGF by BMMSCs was enhanced under hypoxic conditions, we cultured BMMSCs in hypoxic conditions for 24 hours. The supernatant was collected for ELISA of VEGF. The level of VEGF in supernatant of hypoxia-conditioned BMMSCs was significantly higher than in normoxia-conditioned BMMSCs (101.1 ± 12.02 pg/10^5^ cells vs*.* 568.5 ± 34.79 pg/10^5^ cells, *P* <0.01; Figure 2).

### Normoxia-conditioned BMMSCs promoted proliferation of hepatocytes but not liver regeneration and survival

**Figure 3:**
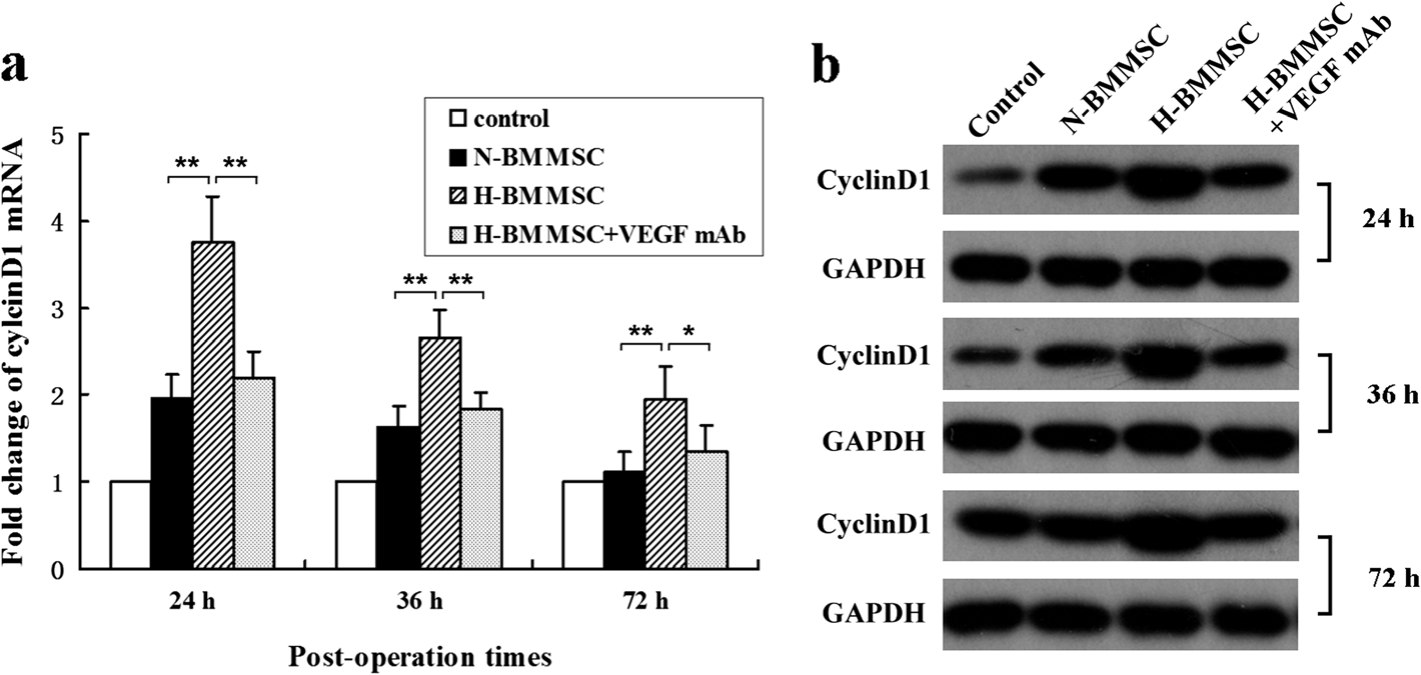
**mRNA and protein levels of cyclin D**_**1 **_**postoperatively. (a)** mRNA level of cyclin D_1_ 24, 36, and 72 hours postoperatively by real-time PCR. **(b)** Protein expression of cyclin D_1_ 24, 36, and 72 hours postoperatively by western blot. *n* = 6, *P <0.05 and **P <0.01. H-BMMSC, hypoxia-conditioned bone marrow mesenchymal stem cells; N-BMMSC, normoxia-conditioned bone marrow mesenchymal stem cells; VEGF, vascular endothelial growth factor.

**Figure 4:**
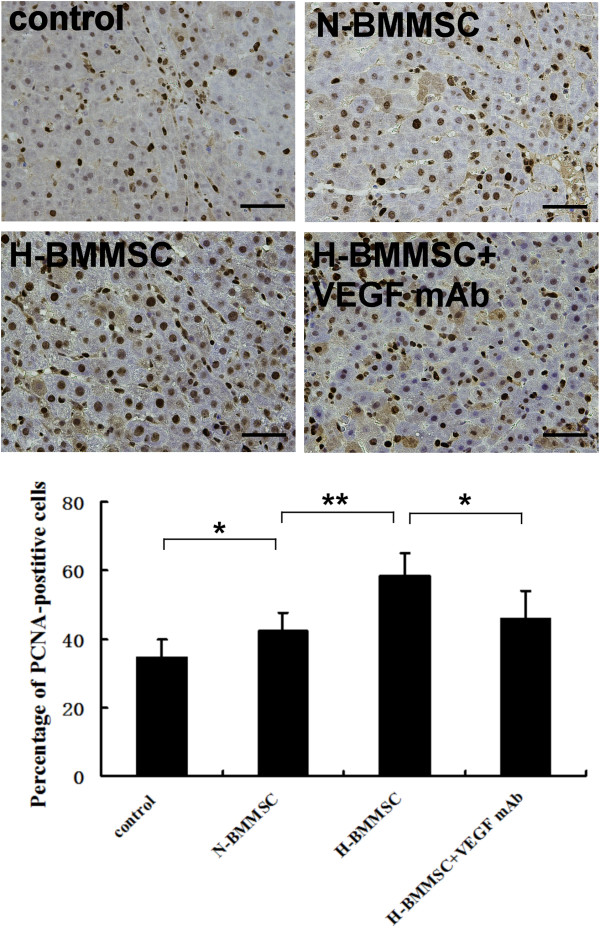
**Proliferating cell nuclear antigen expression in liver slides on postoperative day 3.** Cells with brown nuclei were proliferating cell nuclear antigen (PCNA)-positive. Scale bar represents 20 μm. *n* = 6, *P <0.05 and **P <0.01. H-BMMSC, hypoxia-conditioned bone marrow mesenchymal stem cells; N-BMMSC, normoxia-conditioned bone marrow mesenchymal stem cells; VEGF, vascular endothelial growth factor.

**Figure 5:**
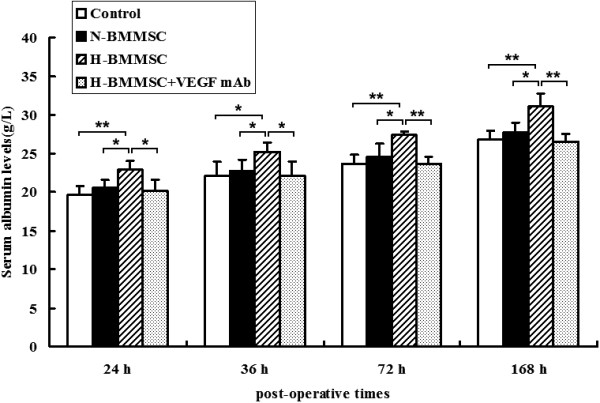
**Serum albumin levels 24, 36, 72, and 168 hours postoperatively.***n* = 6, *P <0.05 and **P <0.01. H-BMMSC, hypoxia-conditioned bone marrow mesenchymal stem cells; N-BMMSC, normoxia-conditioned bone marrow mesenchymal stem cells; VEGF, vascular endothelial growth factor.

**Figure 6:**
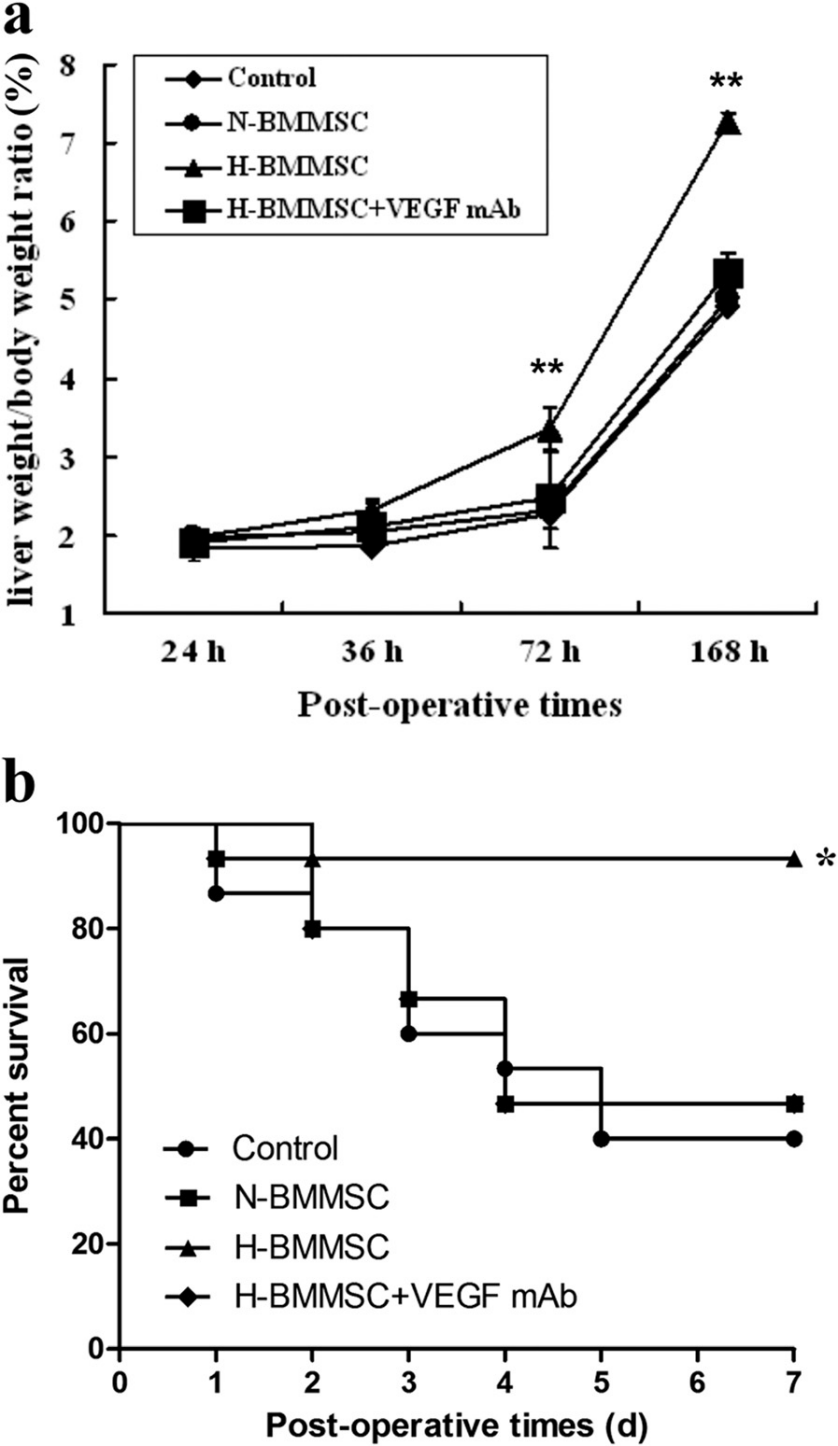
**Remnant liver volume and survival of postoperative animals. (a)** Liver weight/body weight ratios 24, 36, 72, and 168 hours postoperatively. **(b)** Seven-day survival curves of postoperative animals. *n* = 6, *P <0.05 and **P <0.01 when comparing data of the H-BMMSC group with data of the other three groups. H-BMMSC, hypoxia-conditioned bone marrow mesenchymal stem cells; N-BMMSC, normoxia-conditioned bone marrow mesenchymal stem cells; VEGF, vascular endothelial growth factor.

We explored the therapeutic effect of normoxia-conditioned BMMSCs in our massive hepatectomy model. The mRNA and protein levels of cyclin D_1_ mRNA in the N-BMMSC group were significantly higher than those in the control group postoperatively (Figure 3), indicating a higher level of DNA synthesis in the N-BMMSC group. Similarly, the percentage of PCNA-positive cells was higher in the N-BMMSC group than in the control group on postoperative day 3 (42.28 ± 5.288 vs*.* 34.33 ± 5.602, *P* <0.05; Figure 4). However, no significant difference was seen in serum albumin levels (Figure 5) and the liver weight/body weight ratio between these two groups at 24, 36, 72, and 168 hours postoperatively (Figure 6a). In addition, 7-day survival rates were similar between these two groups (40% in control group vs*.* 46.7% in the N-BMMSC group; Figure 6b).

### Hypoxia-conditioned BMMSCs reduced liver injury, and enhanced proliferation of hepatocytes, liver regeneration and survival

To maximize the therapeutic potential of infused BMMSCs, we evaluated the effects of hypoxia preconditioned BMMSCs on liver injury and regeneration in this massive hepatectomy model. We found that the serum level of albumin significantly increased in the H-BMMSC group compared with the N-BMMSC and control groups (Figure 5). The mRNA and protein levels of cyclin D_1_ (Figure 3) and percentage of PCNA-positive cells (Figure 4) were further elevated in the H-BMMSC group compared with those in the N-BMMSC group. In addition, the liver weight/body weight ratio and 7-day survival rate of the H-BMMSC group were significantly higher than that of the N-BMMSC and control groups (Figure 6a,b).

### VEGF mRNA and protein levels were elevated in hypoxia-conditioned BMMSCs

**Figure 7:**
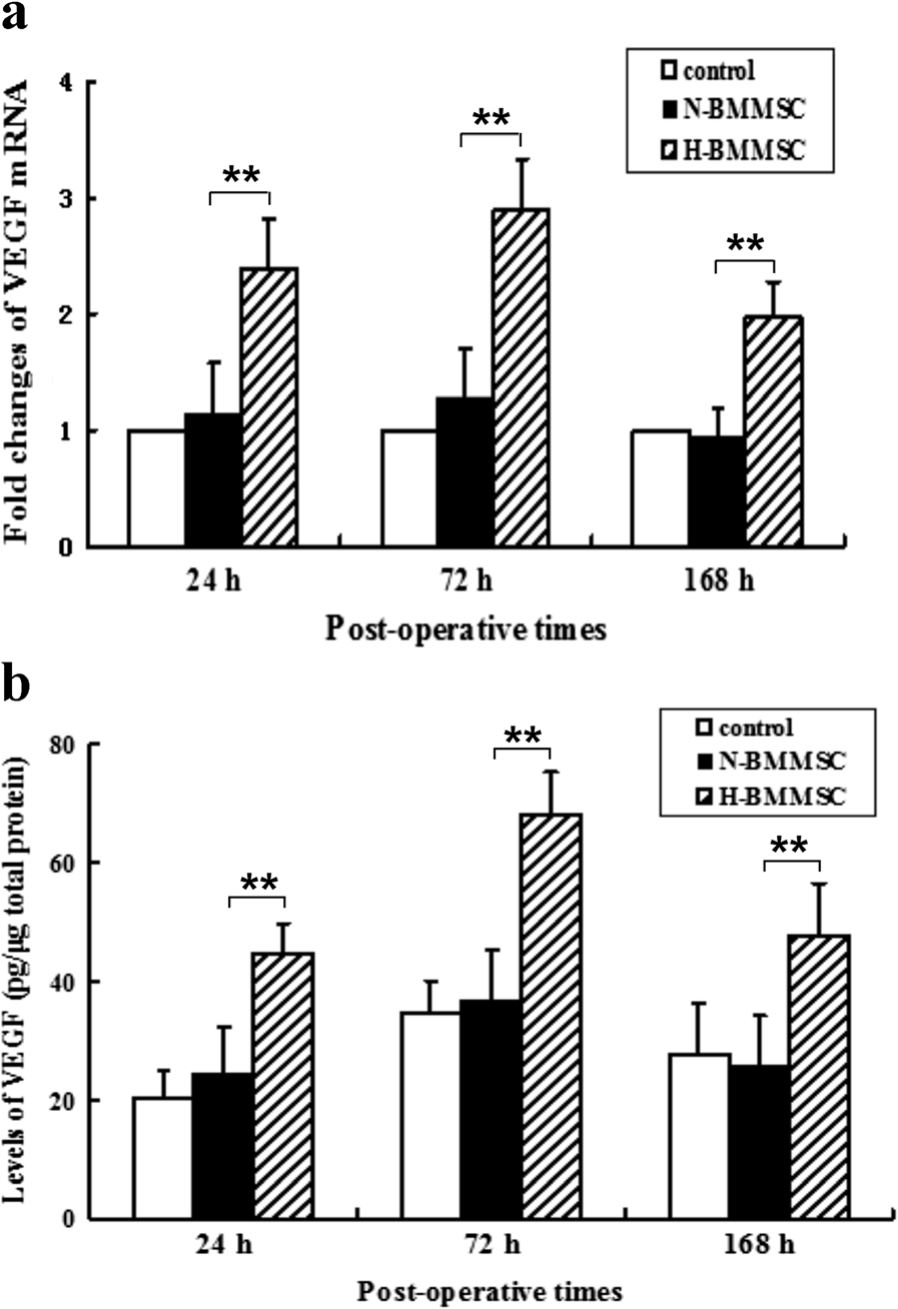
**mRNA and protein levels of vascular endothelial growth factor in rat liver postoperatively. (a)** mRNA level of VEGF 24, 72, and 168 hours postoperatively by real-time PCR. **(b)** Protein expression of VEGF 24, 72, and 168 hours postoperatively by ELISA. *n* = 6, **P <0.01). H-BMMSC, hypoxia-conditioned bone marrow mesenchymal stem cells; N-BMMSC, normoxia-conditioned bone marrow mesenchymal stem cells; VEGF, vascular endothelial growth factor.

The level of VEGF mRNA was much higher in the H-BMMSC group than in the control and N-BMMSC groups 24, 72, and 168 hours postoperatively (Figure 7a). Similarly, the level of VEGF in liver homogenate of the H-BMMSC group was much higher than in the control and N-BMMSC groups 24, 72, and 168 hours postoperatively (Figure 7b).

### Injection of VEGF mAb diminished the therapeutic effect of hypoxia-conditioned BMMSCs

To evaluate the possible involvement of the elevated VEGF in the H-BMMSC therapeutic effect, we injected rat VEGF mAb into perioperative rats. We found after VEGF was inhibited by the antibody that the therapeutic effects of H-BMMSCs were mostly diminished. The expression of cyclin D_1_ (Figure 3), percentage of PCNA-positive cells (Figure 4), liver weight/body weight ratio (Figure 6a) and 7-day survival rate (Figure 6b) in the H-BMMSC + VEGF mAb group all significantly declined compared with the H-BMMSC group. The serum albumin level was significantly lower in the H-BMMSC + VEGF mAb group than in the H-BMMSC group (Figure 5). These results indicated that VEGF may play an important role in H-BMMSC pro-regenerative effects.

## Discussion

BMMSCs are reported to reduce liver injuries and promote liver regeneration in many animal models. The therapeutic effects vary, and the underlying mechanisms are distinct, sometimes controversial. These variations are because animal models used previously were mostly toxic liver injuries using agents such as Carbon tetrachloride and d-galactosamine. The accompanying chemical injuries may affect the process of liver regeneration by altering the release of cytokines and directly inhibiting the proliferation of hepatocytes. Here we introduced a rat model of massive hepatectomy. The regeneration stimuli are adequate, and some degree of liver injury exists.

In this study, we found that infusion of BMMSCs may facilitate the proliferation of hepatocytes after massive hepatectomy in rats, reflected by the elevated expression of cyclin D_1_ and PCNA-positive cells. However, the infusion neither reduces liver injury nor accelerates the process of liver regeneration. These results are similar to reports from our researchers. Hu and colleagues demonstrated that normoxia-conditioned MSCs have little effect on rat infarcted myocardium, but hypoxia preconditioning significantly enhances the capacity of MSCs to reduce cell death and apoptosis, increasing angiogenesis/visualization [24]. The pro-regenerative potential of normoxia-conditioned BMMSCs may therefore be less impressive.

Hypoxia preconditioning for MSCs was first reported by Annabi and colleagues, who found that hypoxia enhances the expression of VEGF and MT1-matrix metalloproteinase and facilitates migration and tube formation of MSCs [20]. More data showed that, after incubating MSCs in hypoxic environments, the biological features of these cells are apparently altered. This alteration is characterized by upregulation of several pro-regenerative, pro-angiogenic, and anti-apoptotic factors such as hypoxia-inducible factor-1α, VEGF and insulin-like growth factor-1 [24-27]. Hypoxia preconditioning was confirmed to be an effective way of enhancing the therapeutic effects of MSCs in many diseases. Rosová and colleagues demonstrate that MSCs enhanced revascularization after surgical induction of hind limb ischemia in mice, and the restoration of blood flow was observed significantly earlier in mice that had been injected with hypoxic preconditioned MSCs [21]. Other researchers also reported similar results in a myocardial infarction model [28] and in a traumatic brain injury model [29].

Here we found that hypoxia-conditioned BMMSCs secreted significantly more VEGF than normoxia-conditioned cells. The levels of VEGF mRNA and protein were also upregulated postoperatively in the H-BMMSC group. Taken together, these results indicated that elevated VEGF might play the key role in the H-BMMSC therapeutic effect in this model. The perioperative application of VEGF mAb successfully blocked the H-BMMSC effects in promoting liver regeneration, further supporting this hypothesis. VEGF is considered an important regulator in liver regeneration. Some studies report that delivery of VEGF increases liver mass *in vivo* but does not promote proliferation of hepatocytes *in vitro*, unless liver sinusoidal endothelial cells are also present, indicating that VEGF promotes liver regeneration via stimulating the proliferation of liver sinusoidal endothelial cells [17]. VEGF treatment can also significantly reduce the mortality rate of rats subjected to acute liver failure through maintenance of liver sinusoidal endothelial cell architecture [30]. Ding and colleagues reported the VEGF-dependent regenerative stimuli are important in inducing proliferation of mature hepatocytes after partial hepatectomy, highlighting the role of VEGF in liver regeneration [18]. Now widely accepted is that liver regeneration is an angiogenesis-dependent phenomenon, and liver sinusoidal endothelial cells are a key mediator of liver regeneration after partial hepatectomy [31,32]. Hence, BMMSC-derived VEGF might promote liver regeneration by enhancing the regeneration of liver sinusoidal endothelial cells and hepatocytes in this model. In addition, the dynamic expression of VEGF after operation in this study further confirmed this hypothesis. We found that the peak of VEGF expression in this study occurred at 72 hours, which coincided with the proliferation of liver sinusoidal endothelial cells in liver regeneration [33].

Although the *in vitro* experiment showed that BMMSCs secreted much more VEGF by hypoxia preconditioning, the *in vivo* upregulation of VEGF may only partially be due to elevated production of VEGF by infused H-BMMSCs. As liver parenchymal cells also produce VEGF and the production might be regulated by surrounding BMMSCs. IL-6 [34], hepatocyte growth factor [18], and insulin-like growth factor-1 [35], which could be secreted by BMMSCs, are reported to promote the production of VEGF. The infused H-BMMSCs might therefore indirectly upregulate the expression of VEGF in liver parenchymal cells. However, the expressions of other factors such as IL-6 and hepatocyte growth factor after hypoxia conditioning need further exploration.

The transdifferentiation potential of infused BMMSCs was not evaluated in this study. The consensus is that the main therapeutic effects of MSCs are their paracrine function of various factors but not differentiating into functional tissue cells. Taking cell fusion into consideration, few, if any, infused MSCs transdifferentiate into functional liver cells [36], and the duration for successful differentiation of MSCs is reported to be at least 2 weeks [37]. Perhaps it is therefore of little value to assess the possible differentiation of infused BMMSCs within 1 week after implantation.

## Conclusion

In summary, we found that the infusion of hypoxia preconditioned BMMSCs reduced liver injury and promoted liver regeneration and survival in a rat massive hepatectomy model. The upregulation of VEGF might mediate the therapeutic effects of infused BMMSCs. Further studies are needed to evaluate the exact cell source of up-expressed VEGF and the mechanisms of its pro-regenerative effects in this model.

## Abbreviations

BMMSC: bone marrow mesenchymal stem cell; BSA: bovine serum albumin; DMEM: Dulbecco’s modified Eagle’s medium; ELISA: enzyme-linked immunosorbent assay; IL: interleukin; mAb: monoclonal antibody; MSC: mesenchymal stem cell; PCNA: proliferating cell nuclear antigen; PCR: polymerase chain reaction; RT: reverse transcriptase; VEGF: vascular endothelial growth factor.

## Competing interests

The authors declare that they have no competing interests.

## Authors’ contributions

JY and SZ conceived, interpreted and analyzed the data and wrote the manuscript. SY and FG carried out the animal experiments. WZ and YL carried out the isolation and culture of BMMSCs, the ELISA assay, and immunohistological analysis. ZC performed the PCR and western blot assay. MZ and JH have been involved in design of the experiments and revising it critically for important intellectual content. All authors read and approved the final manuscript.
